# Medical and Philosophical News

**Published:** 1781-05

**Authors:** 


Dr. Hunter of York, F. R. S. has lately experienced the efficacy of a vapour bath, in a caſe of hydrocephalus internus. The diſeaſe, previous to the trial of this remedy, is ſaid to have have been confiderably advanced, the fight of the right eye having been much affected, and the ufe of the child's limbs almoft totally loft. By perfevering in the ufe of this bath a fhort time, the patient was cured.—In another cafe of this kind, a refpectable phyfician in London has feen favourable effects take place from the ufe of calomel. The child took a grain of it daily; when the mouth began to be affected, it was difcontinued for a few days, and afterwards had recourfe to again. This courfe was purfued, till the patient had taken about thirty grains of calomel. During all this time the progrefs of the difeafe feemed at leaft to be fufpended by the remedy, as the child's head meafured eighteen inches in circumference previous to the exhibition of the mercury, and there was no apparent increafe in its bulk during the mercurial courfe. But at length the parents of the child becoming unwilling to try it any longer, it was difcontinued, and fince that time the head has gradually increafed in fize, meafuring now about twenty-two inches in circumference.

